# Curcumin interacts directly with the Cysteine 259 residue of STAT3 and induces apoptosis in H-*Ras* transformed human mammary epithelial cells

**DOI:** 10.1038/s41598-018-23840-2

**Published:** 2018-04-23

**Authors:** Young-Il Hahn, Su-Jung Kim, Bu-Young Choi, Kyung-Cho Cho, Raju Bandu, Kwang Pyo Kim, Do-Hee Kim, Wonki Kim, Joon Sung Park, Byung Woo Han, Jeewoo Lee, Hye-Kyung Na, Young-Nam Cha, Young-Joon Surh

**Affiliations:** 1 Tumor Microenvironment Research Center and Research Institute of Pharmaceutical Science, Seoul National University, Seoul, 08826 South Korea; 2 Department of Pharmaceutical Science and Engineering, School of Convergence Bioscience and Technology, Seowon University, Chungbuk, 361-742 South Korea; 3 Department of Applied Chemistry, Institute of Natural Science, Global Center for Pharmaceutical Ingredient Materials, Kyung Hee University, Yongin, 17104 South Korea; 4 Department of Food Science and Biotechnology, College of Knowedge-based Services Engineering, Sungshin Women’s University, Seoul, 02844 South Korea; 5 Inha University, College of Medicine, Incheon, South Korea

## Abstract

Signal transducer and activator of transcription 3 (STAT3) is a transcription factor that is latent but constitutively activated in many types of cancers. It is well known that STAT3 plays a key role in inflammation-associated tumorigenesis. Curcumin is an anti-inflammatory natural compound isolated from the turmeric (*Curcuma longa* L., Zingiberaceae) that has been extensively used in a traditional medicine over the centuries. In the present study, we have found that curcumin inhibits STAT3 signaling that is persistently overactivated in H-*Ras* transformed breast epithelial cells (H-*Ras* MCF10A). Specific cysteine residues present in STAT3 appear to be critical for the activity as well as conformation of this transcription factor. We identified the cysteine residue 259 of STAT3 as a putative site for curcumin binding. Site-directed mutation of this cysteine residue abolished curcumin-induced inactivation of STAT3 and apoptosis in H-*Ras* MCF10A cells. The α,β-unsaturated carbonyl moiety of curcumin appears to be essential in its binding to STAT3 in H-*Ras* MCF10A cells. Tetrahydrocurcumin that lacks such electrophilic moiety failed to interact with STAT3 and to induce apoptosis in the same cell line. Taken together, our findings suggest that curcumin can abrogate aberrant activation of STAT3 through direct interaction, thereby inhibiting STAT3-mediated mammary carcinogenesis.

## Introduction

Transcription factors that belong to the family of Signal Transducer and Activator of Transcription (STAT) are activated by various cytokines, growth factors and hormones^1–3^. STATs exist as latent monomers in the cytoplasm. However, upon ligand stimulation, STATs become phosphorylated at a single carboxyterminal tyrosine by receptor-intrinsic, receptor-associated, or nonreceptor tyrosine kinases^1,2^. Subsequently, phosphorylated STATs undergo dimerization through reciprocal interaction between the Src Homology 2 (SH2) domain and the phosphorylated tyrosine residue^4,5^. STAT dimers then translocate to nucleus where they bind to the promoters of target genes^6^. STAT3 is one of the STAT family members and known to play a key role in inflammation-associated tumorigenesis^1,7^. In normal cells, STAT3 signaling is transiently activated, and its activation is tightly regulated by extracellular stimuli. However, aberrant STAT3 activation leads to cancer cell proliferation, survival and resistance to apoptosis, thereby accelerating tumor development and progression^5,8^. Conversely, disruption of STAT3 signaling by antisense oligonucleotides or STAT3 mutants results in growth inhibition and induction of apoptosis in various types of cancer cells^9^. Therefore, STAT3 is considered to be an attractive target for cancer prevention and therapy.

Curcumin is a natural polyphenolic compound isolated from turmeric (*Curcuma longa*), a spice that has been widely used in a traditional medicine for many centuries^10^. This compound is known to exert anti-cancer effects through regulation of a variety of intracellular signaling pathways and protein-protein interactions^11^. Although, curcumin has the ability to inhibit JAK/STAT signaling^12–14^, the molecular mechanism by which curcumin inactivates STAT3 remains elusive. Notably, curcumin possesses an α, β unsaturated carbonyl moiety and can act as an electrophile^15^. This prompted us to investigate whether curcumin could effectively inhibit aberrantly activated STAT3 and induce apoptosis in H-*Ras* transformed human mammary epithelial (H-*Ras* MCF10A) cells by directly interacting with this transcription factor.

## Results

### Curcumin induces apoptosis in H-*Ras* MCF10A cells

**Figure 1:**
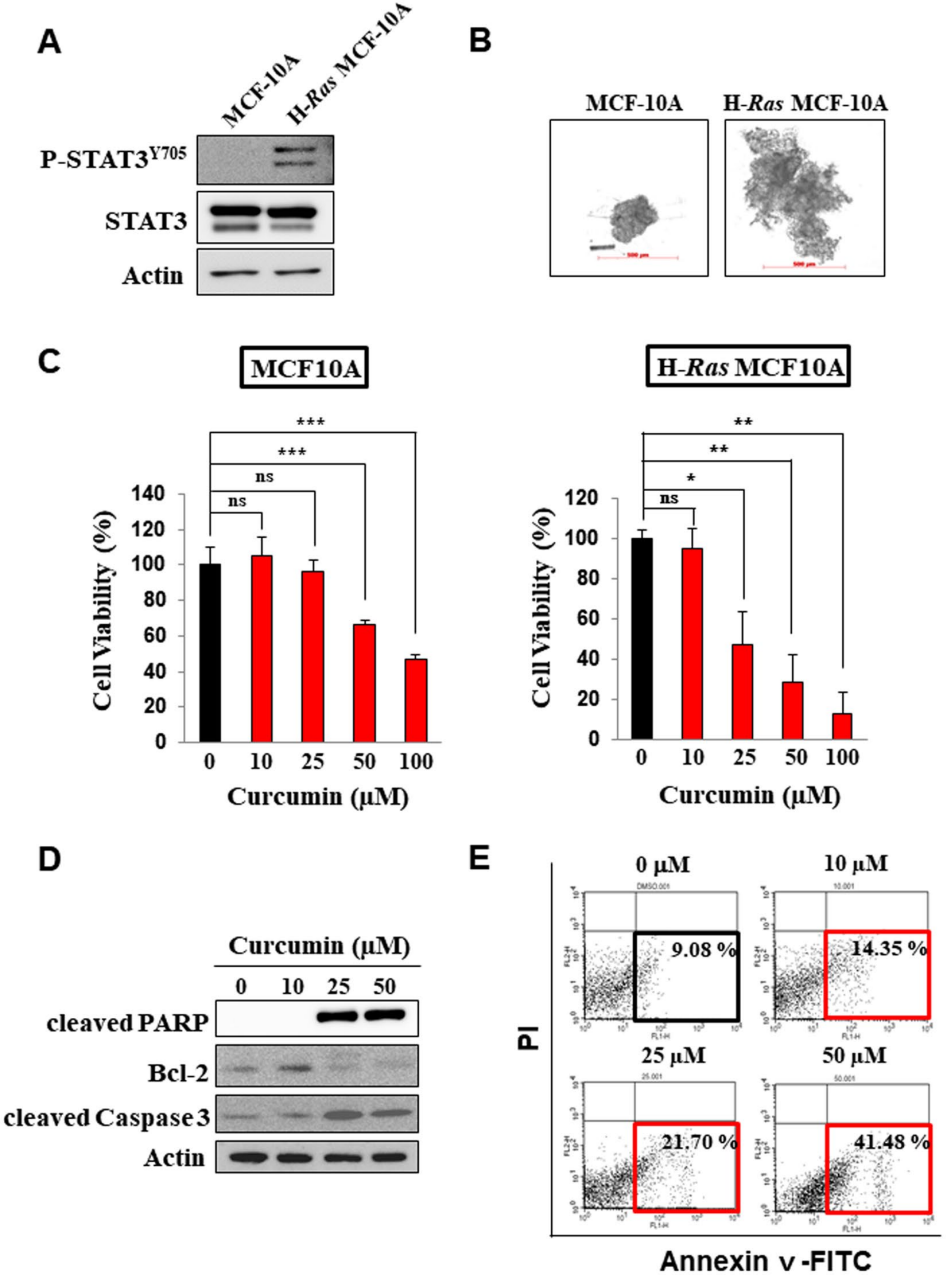
Comparison between MCF10A and H-*Ras* MCF10A cells and the effect of curcumin on induction of apoptosis. (**A**) Total cell lysates of MCF10A and H-*Ras* MCF10A cells were prepared, and the expression of phosphorylated STAT3 was assessed by Western blot analysis. (**B**) The same number of MCF10A and H-*Ras* MCF10A cells (2 × 10^3^) was seeded onto Lipidure®-Coat Plate A-U96 and incubated for 5 days. The spheroid formations were observed under the microscope. scale bar, 500 μm. Data are means ± standard deviation (SD). ns: not significant; *P < 0.05; **P < 0.005; ***P < 0.001. (**C**) The MTT assay was conducted with MCF10A and H-*Ras* MCF10A cells treated with various concentrations of curcumin for indicated time. Data are means ± SD (*P < 0.05). (**D**) H-*Ras* MCF10A cells were treated for 24 h with indicated concentrations of curcumin. The expression of cleaved PARP, Bcl-2 and cleaved caspase 3 was observed by Western blot analysis. (**E**) H-*Ras* MCF10A cells were treated with curcumin (10 μM, 25 μM or 50 μM) for 24 h and then incubated with Annexin V and PI for 15 min. The proportion of apoptotic cells was measured by FACS analysis.

**Figure 2:**
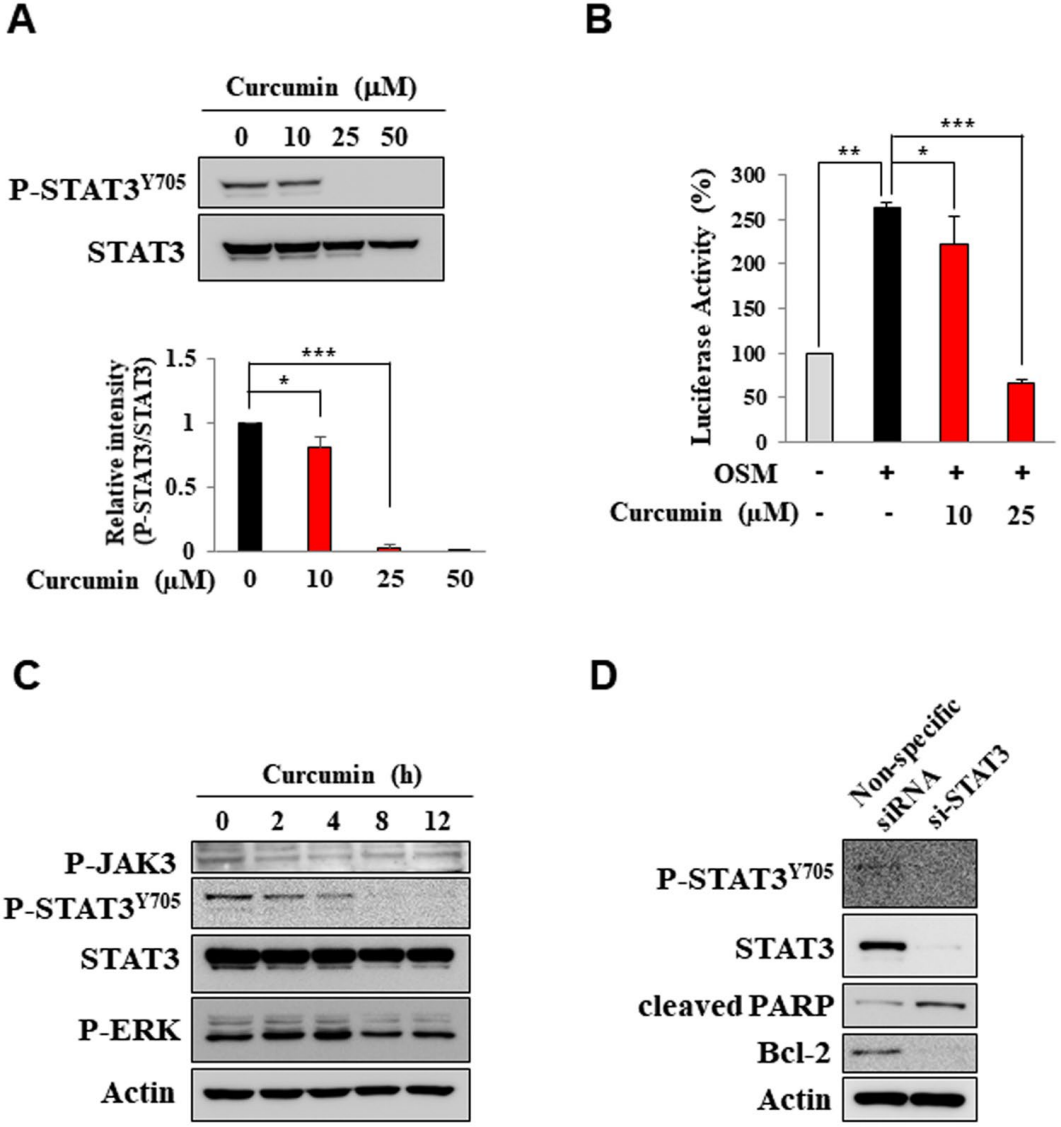
Curcumin-induced apoptosis of H-*Ras* MCF10A cells through inhibition of STAT3 signaling. (**A**) H-*Ras* MCF10A cells were treated with indicated concentrations of curcumin for 24 h. The expression of phosphorylated STAT3 was assessed by Western blot analysis. Actin was used as an equal loading control. *P < 0.05; ***P < 0.001. (**B**) Luciferase activity was measured with HeLa/P-STAT3-luc cells preincubated with indicated concetrations of curcumin for 12 h and then stimulated with OSM for 6 h. *P < 0.05; **P < 0.005; ***P < 0.001. (**C**) H-*Ras* MCF10A cells were incubated with curcumin (25 μM) for indicated time periods. The expression of phosphorylated STAT3 and upsteam kinases was determined by Western blot analysis. (**D**) H-*Ras* MCF10A cells were treated with scrambled or STAT3 siRNA for 48 h. PARP cleavage and Bcl-2 expression were detected by Western blot analysis.

STAT3 has been reported to play a key role in anti-apoptosis and proliferation of cancerous and transformed cells^7,9,16^. H-*Ras* MCF10A cells have a constitutively elevated level of activated STAT3 compared with normal human mammary epithelial MCF10A cells (Fig. 1A) and grow faster and more aggressively (Fig. 1B). Notably, H-*Ras* MCF10A cells were found to be more susceptible to curcumin-induced cytotoxicity than non-oncogenic MCF10A cells (Fig. 1C). The anti-proliferative activity of curcumin appears to be associated with its induction of apoptosis as evidenced by cleavage of poly(ADP-ribose) polymerase (PARP), down-regulation of the antiapoptotic protein Bcl-2 (Fig. 1D) and an increased proportion of annexin V-positive and propidium iodide (PI)-negative cells (Fig. 1E). When incubated with H-*Ras* MCF10A cells, curcumin at 25 μM completely inhibited the phosphorylation of STAT3, an essential event in the activation of this transcription factor (Fig. 2A). To assess the effect of curcumin on STAT3 transcriptional activity, the luciferase reporter gene assay was performed. Consistent with its suppression of phosphorylation of STAT3, curcumin inhibited the STAT3 transcriptional activity in oncostatin M (OSM)-stimulated HeLa cells harboring P-STAT3-luc reporter construct (Fig. 2B). However, curcumin had little effects on the activation of Janus kinase 3 (JAK3) and extracellular signal-regulated kinase (ERK) responsible for STAT3 phosphorylation at tyrosine 705 and serine 727 residues, respectively (Fig. 2C). This finding suggests that curcumin may directly target STAT3 without modulating the upstream kinases. In another experiment, H-*Ras* MCF10A cells were transfected with STAT3 siRNA. Silencing of *STAT3* gene resulted in cleavage of PARP and downregulation of Bcl-2 (Fig. 2D), suggesting that curcumin induces apoptosis, at least in part, by targeting STAT3 signaling.

### Tetrahydrocurcumin, a non-electrophilic analog of curcumin, fails to inhibit STAT3 signaling and to induce apoptosis

**Figure 3:**
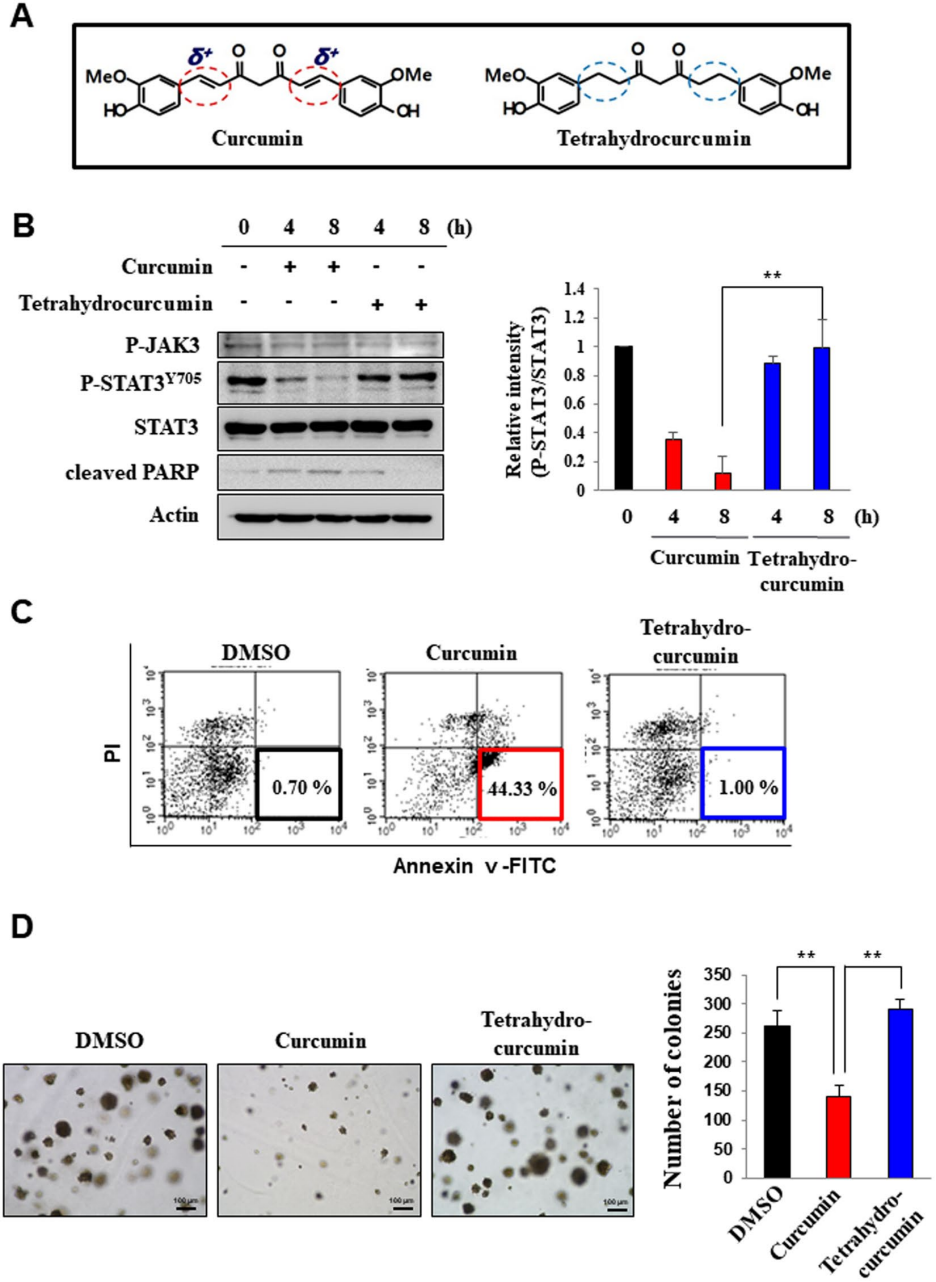
Involvement of the α,β unsaturated carbonyl group of curcumin in its inhibition of STAT3 activation and induction of apoptosis. (**A**) Curcumin has the two α, β unsaturated carbonyl groups which are eliminated in tetrahydrocurcumin by hydrogenating the double bonds. (**B**) H-*Ras* MCF10A cells were incubated with vehicle, curcumin (25 μM) or tetrahydrocurcumin (25 μM). The expression of phosphorylated STAT3, STAT3 and related kinase was determind by Western blot analysis. (**C**) H-*Ras* MCF10A cells were treated with curcumin or tetrahydrocurcumin (25 μM each) for 12 h and then incubated with Annexin V and PI for 15 min. The population of apoptotic cells was measured by FACS analysis. (**D**) H-*Ras* MCF10A cells were cultured in soft agar for 10 days. Curcumin or tetrahydrocurcumin was added to the medium every other day for another 10 days. The colony formation was visualized under the microscope. **P < 0.005. scale bar, 100 µm.

The interaction between an electrophile and a nucleophile causes the formation of a covalent bond that provides a link for the two entities^17^. It has been suggested that the modification of cysteine thiols can largely affect the 3 dimensional structure of many redox sensitive proteins and their functions^15^. One of the structural characteristics of curcumin is presence of the α,β-unsaturated carbonyl moiety^17^. In order to determine whether this electrophilic nature of curcumin is essential for its suppression of STAT3 signaling and proapoptotic activity, we utilized tetrahydrocurcumin generated by catalytic hydrogenation of the double bond of curcumin (Fig. 3A). In agreement with the previous data (Figs 1D, 1E and 2A), curcumin treatment significantly reduced the levels of phosphorylated STAT3 and induced PARP cleavage in H-*Ras* MCF10A cells (Fig. 3B), resulting in apoptosis (Fig. 3C). However, its non-electrophilic analogue, tetrahydrocurcumin, barely inhibited both events. In another experiment, we compared the effect of curcumin and tetrahydrocurcumin on anchorage-independent growth of H-*Ras* MCF10A cells. Again, curcumin, but not tetrahydrocurcumin, significantly decreased the number of colonies (Fig. 3D).

### Curcumin, but not tetrahydrocurcumin, inhibits dimerization, subsequent DNA binding and transcriptional activity of STAT3

**Figure 4:**
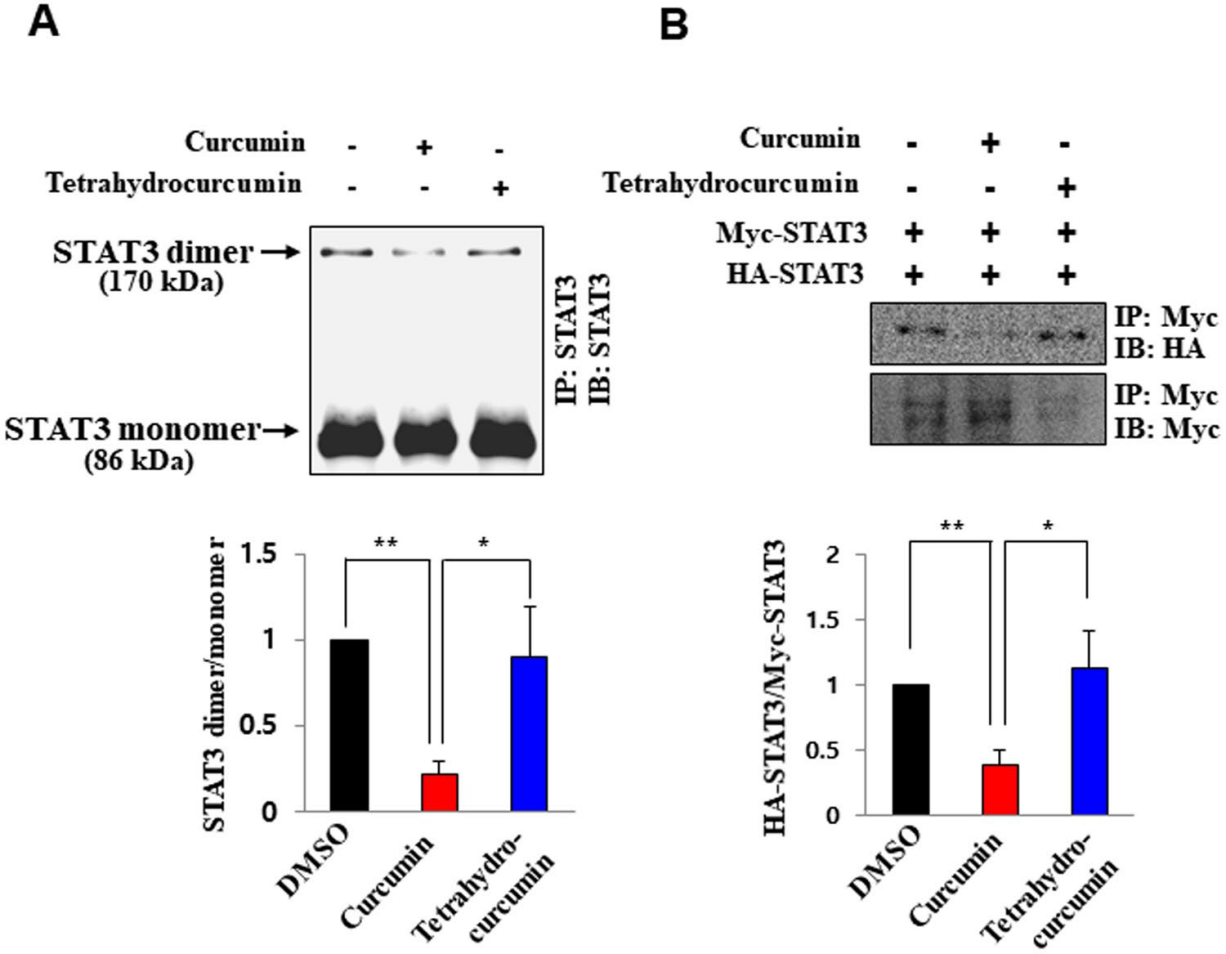
The comparative effect of curcumin and tetrahydrocurcumin on STAT3 dimerization. (**A**) Total lysate from H-*Ras* MCF10A cells treated with curcumin (25 μM) for 12 h was immunoprecipitated with Protein A/G agarose and anti-STAT3 antibody overnight and analyzed by immunobloting with anti-STAT3 antibody. *P < 0.05; **P < 0.005. (**B**) PC-3 cells were co-transfected with HA-tagged STAT3 and Myc-tagged STAT3 and treated with curcumin or tetrahydrocurcumin (25 μM each) for 12 h. The total lysates obtained from the transfected cells were immunoprecipitated with anti-Myc antibody and analyzed by Western blotting with anti-HA antibody. *P < 0.05; **P < 0.005.

**Figure 5:**
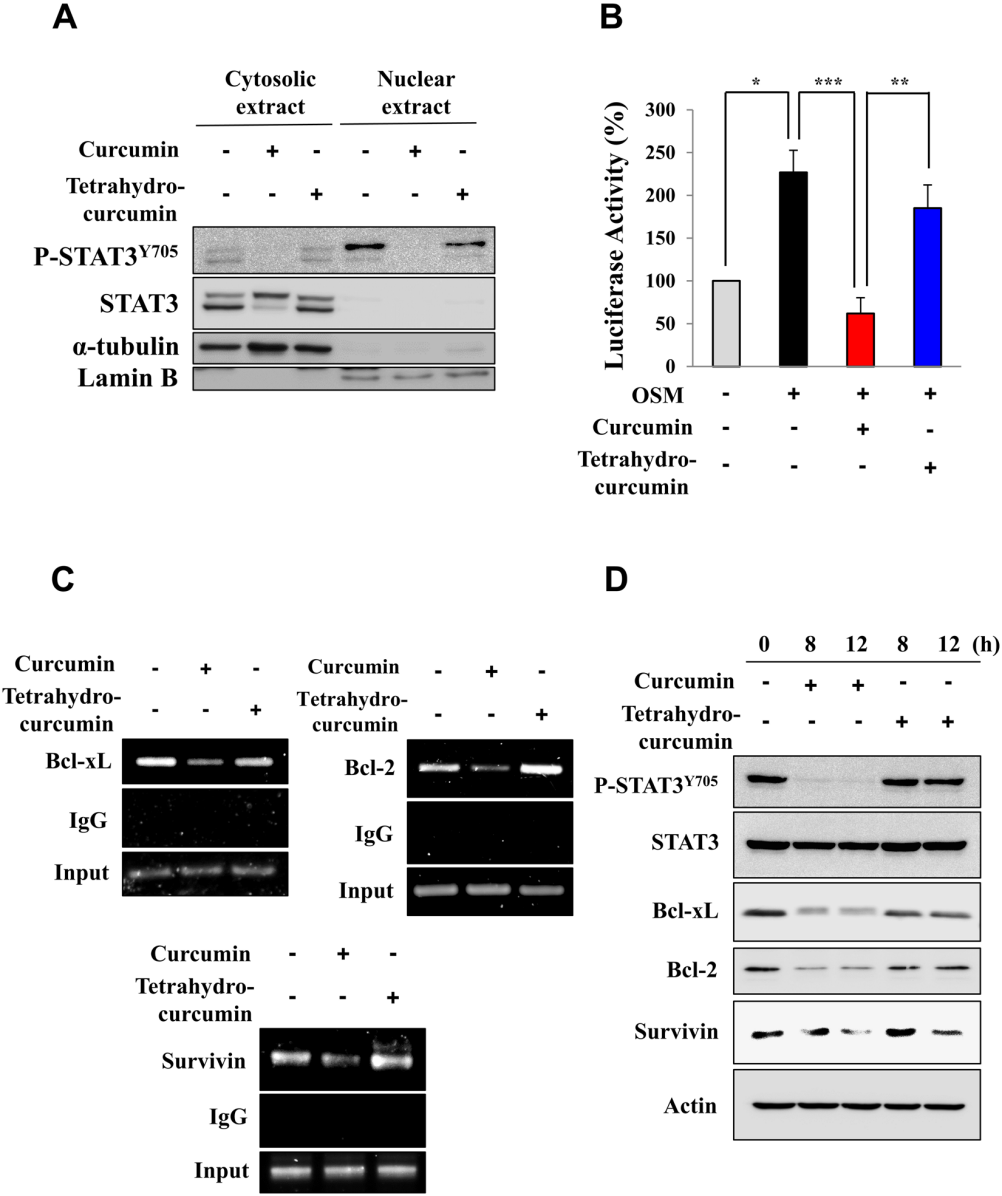
The effect of curcumin and tetrahydrocurcumin on phosphorylation, nuclear translocation, and transcriptional activity of STAT3. (**A**) H-*Ras* MCF10A cells were treated with curcumin and tetrahydrocurcumin (25 μM each) for 12 h. Cytosolic and nuclear fractions were extracted and subjected to Western blot analysis for measuring total and phosphorylated STAT3 levels. (**B**) Luciferase activity was measured in HeLa/P-STAT3-luc cells preincubated with curcumin or tetrahydrocurcumin (25 μM each) for 12 h and then stimulated with OSM for additional 6 h. *P < 0.05; **P < 0.005; ***P < 0.001. (**C**) A chromatin immunoprecipitation (ChIP) assay was conducted using chromatin prepared from H-*Ras* MCF10A cells treated with curcumin or tetrahydrocurcumin (25 μM each) for 12 h. The binding of STAT3 at the promoter of its target genes (*Survivin*, *Bcl-2* and *Bcl-xL*) was determined. (**D**) H-*Ras* MCF10A cells were treated with curcumin or tetrahydrocurcumin (25 μM each) for 8 h or 12 h. The expression of Bcl-2, Bcl-xL and Survivin was analyzed by Western blot analysis.

STAT3 undergoes dimerization through its SH2 domain upon phosphorylation at the tyrosine 705 residue and subsequently translocates to the nucleus^2^. We found that endogenous STAT3 dimerization was inhibited by curcumin, but not by tetrahydrocurcumin (Fig. 4A). We also confirmed that curcumin significantly lowered the level of a dimer formed by exogenously introduced HA-tagged STAT3 and Myc-tagged STAT3, whereas tetrahydrocurcumin was unable to inhibit the formation of an exogenous STAT3 dimer (Fig. 4B). STAT3 nuclear translocation is crucial for its role as a transcription factor^18^. Upon curcumin treatment, nuclear localization of STAT3 was blocked, but this effect was not observed in tetrahydrocurcumin-treated cells (Fig. 5A). STAT3 regulates transcription of genes that encode anti-apoptotic proteins, such as Bcl-xL, Bcl-2 and Survivin^1,2^. Likewise, curcumin, but not tetrahydrocurcumin, significantly inhibited STAT3 luciferase activity (Fig. 5B) as well as the binding to the promoter of STAT3 target genes including *Survivin*, *Bcl-2* and *Bcl-xL* (Fig. 5C). The protein expression of Bcl-xL, Bcl-2 and Survivin was suppressed by curcumin, but such effect was not achieved with its nonelectrophilic analogue, tetrahydrocurcumin (Fig. 5D).

### Curcumin exerts its inhibitory effect on STAT3 signaling through thiol modification

**Figure 6:**
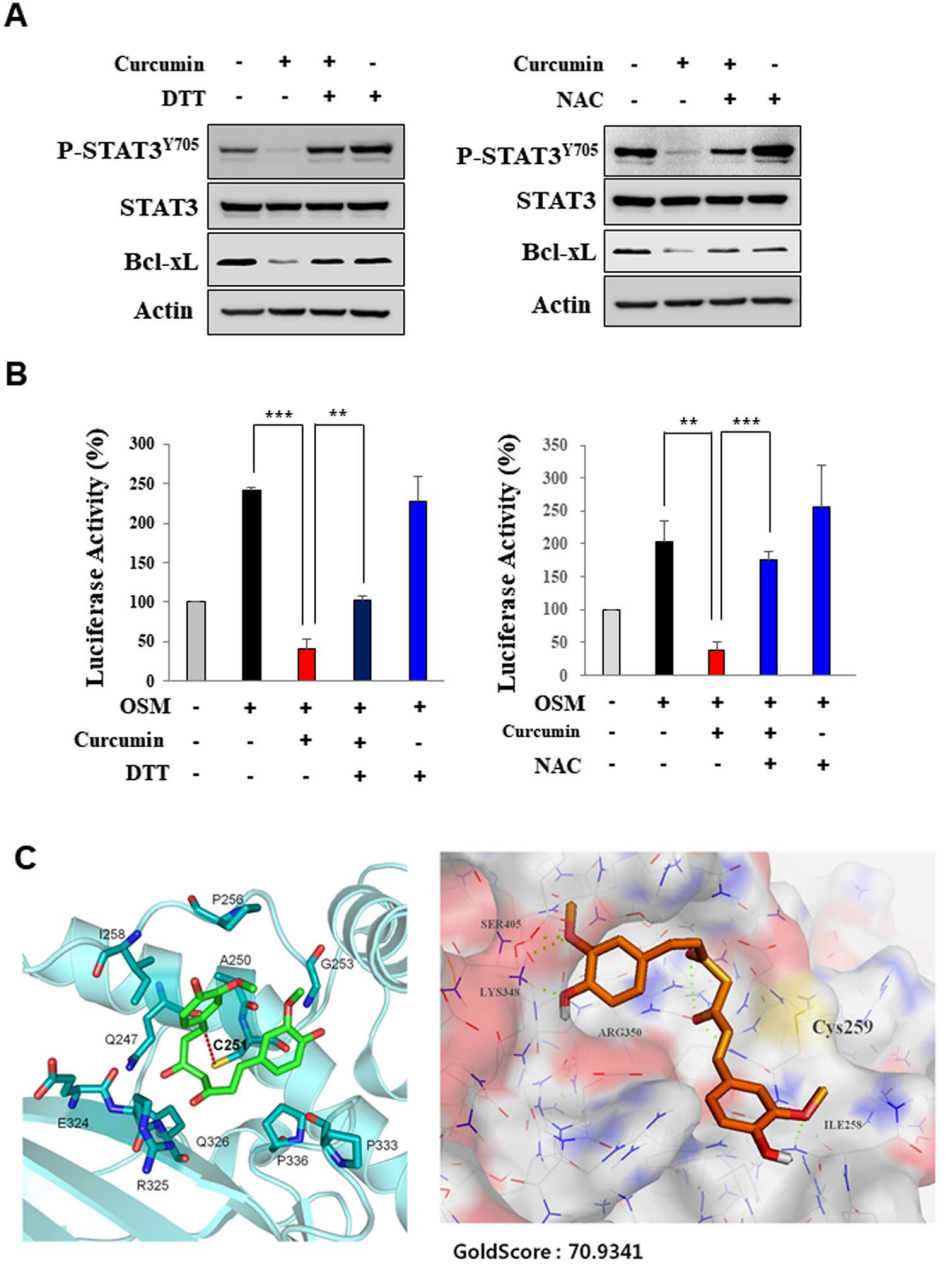
STAT3 thiol modification by curcumin. (**A**) H-*Ras* MCF10A cells were treated with curcumin (25 μM) in the presence or absence of NAC (10 mM) or DTT (0.5 mM). (**B**) Luciferase activity was measured with HeLa/P-STAT3-luc cells preincubated with curcumin (25 µM) in the presence or absence of NAC (10 mM) or DTT (0.5 mM) for 12 h and then stimulated with OSM for another 6 h. **P < 0.005; ***P < 0.001. (**C**) Covalent binding of curcumin to STAT3 as predicted by computational modeling. AutoDock Vina program and GOLD5.0 docking program were used in docking experiments for cysteine 251 and 259, respectively.

As tetrahydrocurcumin lacking the α,β-unsaturated carcbonyl functional group had little effect on suppression of STAT3 signaling, we speculated that the cysteine sulfhydryl group(s) present in STAT3 may be covalently modified by the electrophilic moiety of curcumin. In support of this speculation, the thiol reducing agents, dithiothreitol (DTT) and N-acetylcysteine (NAC), blunted curcumin-mediated inhibition of STAT3 phosphorylation and Bcl-xL expression (Fig. 6A) and transcriptional activity of STAT3 (Fig. 6B). These results suggest that curcumin inhibits STAT3 activation, at least in part, through direct interaction with STAT3, most preferentially at the cysteine residue(s).

### Curcumin may directly modify cysteine 259 in STAT3

**Figure 7:**
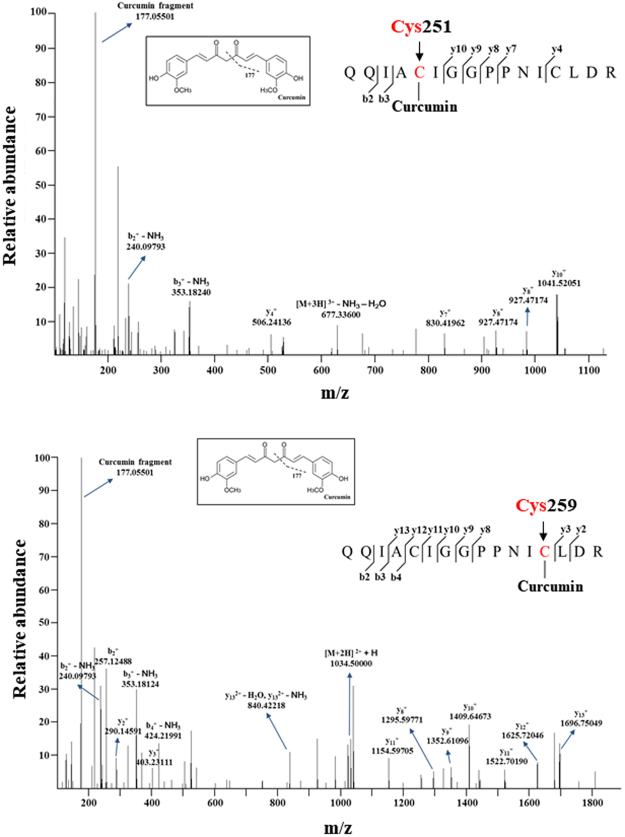
Identification of curcumin binding sites in recombinant STAT3 by LC-MS/MS. Annotated mass spectrum illustrating the binding of curcumin to STAT3 at cysteine 251 and 259 residues was observed. MS/MS spectrum of precursor ion at m/z 1033.49304 [M + 2 H]^+2^ corresponds to the amino acid sequence of STAT3. The elucidation of fragment ion at m/z 177.05501 from the structure was given in the inset of the spectra. The sample preparation and other experimental details for mass spectral analysis are described in Methods.

**Figure 8:**
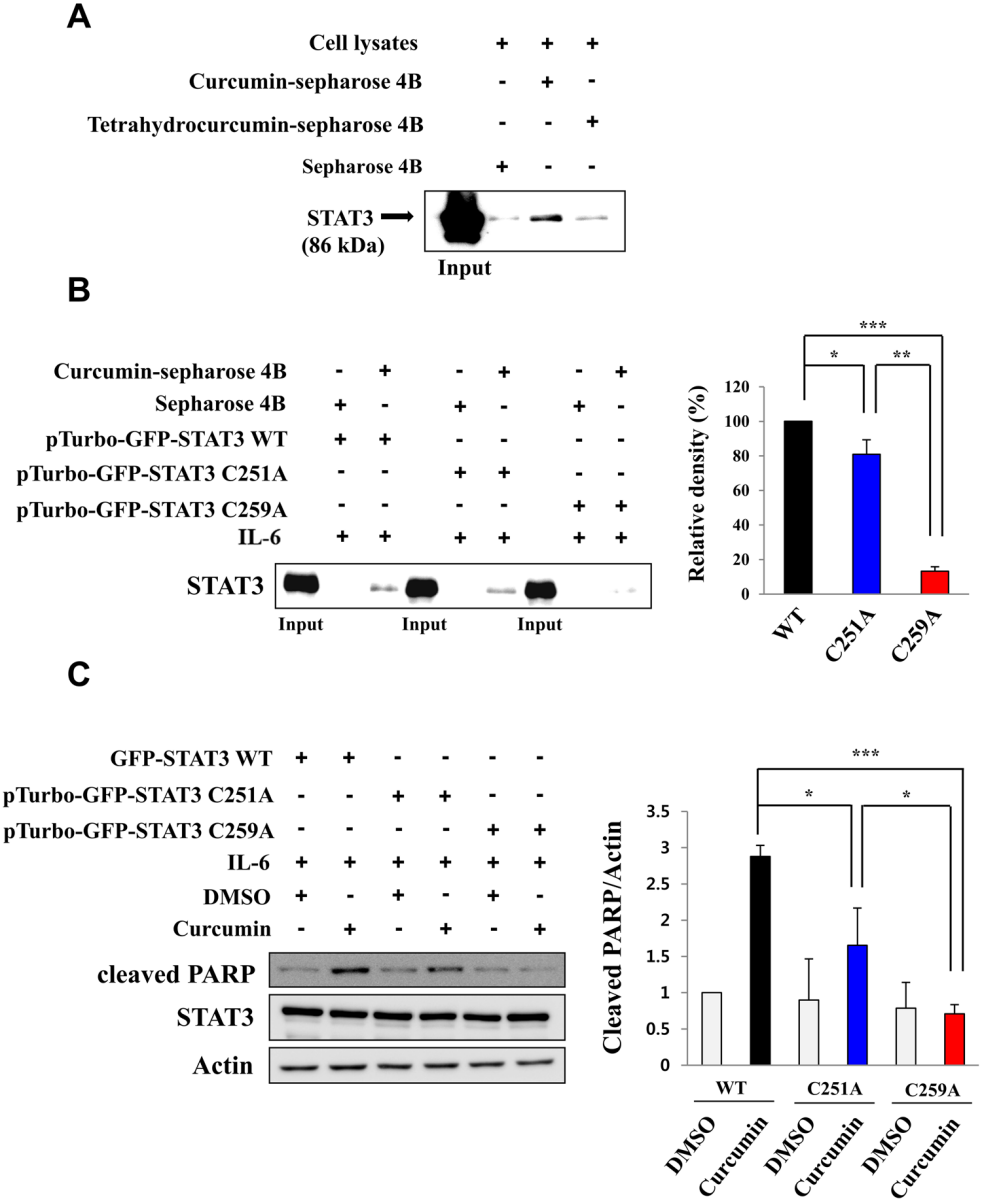
Direct interaction of curcumin with endogenous STAT3 and the cysteine 259 residue of STAT3 as a putative binding site. (**A**) Curcumin-conjugated Sepharose-4B and tetrahydrocurcumin-conjugated Sepharose-4B were incubated with H-*Ras* MCF10A cell lysates overnight. The STAT3 bound to the curcumin- and tetrahydrocurcumin-conjugated Sepharose 4B beads was pulled down by centrifugation and detected by Western blot analysis. (**B**) PC-3 cells transiently transfected with GFP-tagged WT STAT3, GFP-tagged C251A or GFP-tagged C259A were stimulated with IL-60 (50 ng/ml) for 12 h. The cell lysates were incubated with curcumin-conjugated Sepharose-4B. WT STAT3 and STAT3 mutants were detected by anti-STAT3 antibody. *P < 0.05; **P < 0.005; ***P < 0.001. (**C**) PC3 cells were transiently transfected with GFP-tagged WT STAT3, or GFP-tagged mutant STAT3 (STAT-C251A or C259A) and then treated with IL-6 (50 ng/ml) for 12 h, followed by curcumin treatment (25 µM) for 12 h. The expression of cleaved PARP was determined by Western blot analysis. *P < 0.05; **P < 0.005; ***P < 0.001.

STAT3 bears several cysteine residues. Among these, cysteine 259 is surface exposed and important in the dimer formation^19^. Moreover, mutation of STAT3 at cysteine 259 has been reported to abolish the effect of *S*-nitrosoglutathione on interleukin (IL-6)-induced STAT3 phosphorylation and transactivation^20^. *In silico* modeling reveals that curcumin has great potential to bind to STAT3 on cysteine 251 as well as cysteine 259 residue (Fig. 6C). This possibility was further tested by the mass-spectral analysis of STAT3 modified with curcumin. For this purpose, curcumin-treated recombinant STAT3 was subjected to LC-MS/MS. The identified amino acid sequence coverage of the STAT3 protein is indicated in green color (Supplementary Fig. 1A). Two cysteine sites were identified by tandem MS with a high score XCorr value, which include cysteine 251 (XCorr value; 3.12) and cysteine 259 (XCorr value; 2.55) (Fig. 7). The assignments of curcumin binding sites were determined by examining the tandem mass spectra. The diagnostic and most prominent fragment ion at m/z 177.05501 was expected to be derived from curcumin^21^. We also investigated the direct interaction between curcumin and endogenous STAT3 by utilizing CNBr-activated-Sepharose-4B beads. STAT3 protein derived from H-*Ras* MCF10A cells was pulled down with curcumin– or tetrahydrocurcumin-Sepharose 4B beads. As shown in Fig. 8A, curcumin interacted directly with STAT3 but tetrahydrocurcumin did not. To determine whether curcumin modification of cysteine 251 and 259 contributes to inactivation of STAT3, site-directed mutagenesis was conducted in which each of these cysteine residues was replaced by alanine. Curcumin didn’t bind to cysteine 259-mutated STAT3 whilst cysteine 251 mutation barely affected their interaction (Fig. 8B), suggesting that cysteine 259 modification by curcumin is important in its inactivation of STAT3. Apoptosis, in terms of PARP cleavage, was significantly induced by curcumin when PC3 cells were transiently transfected with wild type or cysteine 251-mutated STAT3 (Fig. 8C). Curcumin induced apoptosis, in terms of PARP cleavage, in PC3 cells transiently transfected with wild type STAT3 and to a lesser extent in PC3 cells harbouring cysteine 251-mutated STAT3 (Fig. 8C). However, curcumin-induced apoptosis was abolished in PC3 cells with cysteine 259-mutated STAT3 (Fig. 8C). These findings, taken together, suggest that cysteine 259 of STAT3 would be a *bona fide* target of curcumin in exerting its anti-apoptotic effects.

## Discussion

As a signaling hub onto which many oncogenic signals converge, STAT3 represents an attractive potential target to render tumor cells susceptible to growth inhibition or apoptosis. There are various emerging strategies for targeting STAT3. Direct inactivation of STAT3 probably is one plausible way. There are numerous STAT3 inhibitors including natural compounds. As one of the well-known chemopreventive agents, curcumin has been found to exert its anti-inflammatory and anti-carcinogenic effects by targeting STAT3 signaling^22,23^. However, the mechanism underlying its inactivation of STAT3 has not been well understood.

Owing to the presence of an electrophilic α,β-unsaturated carbonyl moiety, curcumin tends to react with a variety of electron donors. Especially, cysteine thiol residues ubiquitously present in cellular proteins are subject to covalent modification by electrophiles. One of the well-characterized electrophilic and oxidative stress sensing proteins is Kelch-like ECH-associated protein 1 (Keap1). Covalent modification of some highly reactive regulatory cysteine residues in Keap1 drives the activation of Nrf2 signaling^17^. Many studies suggest that electrophile attack of a nucleophile has been considered an important biological process in modulation of cell signaling pathways. One example is 15-deoxy-Δ^12,14^-prostaglandin J_2_, an endogenously produced electrophilic lipid mediator that can potently inhibit NF-κB signaling by binding to cysteine 38 in the p65^24^ and cysteine 62 in the p50 subunits^24,25^ of this transcription factor. Likewise, curcumin has been reported to mediate Michael addition of sulfhydryl groups in diverse cellular proteins, such as inflammatory molecules, cell survival proteins, protein kinases and various enzymes^26^. However, the ability of curcumin to modify STAT3 cysteine residue(s) had not been reported. STAT3 activation has also been found to be sensitive to redox regulation^27^. In line with this notion, cysteine modification, such as *S*-glutathionylation of STAT3 at cysteine 328 and 542, was found to modulate STAT3 signaling^28^.

Considering electrophilic nature of curcumin, thiol modification of STAT3 could account for its inactivation of this transcription factor. Our results show that the thiol reducing agents DTT and NAC restored the phosphorylation and transcriptional activity of STAT3 which were suppressed by curcumin, lending support to the above supposition. In line with this notion, curcumin abrogated phosphorylation, dimerization, DNA binding and transcriptional activity, while tetrahydrocurcumin lacking the electrophilic moiety was incapable of inhibiting all these events.

**Figure 9:**
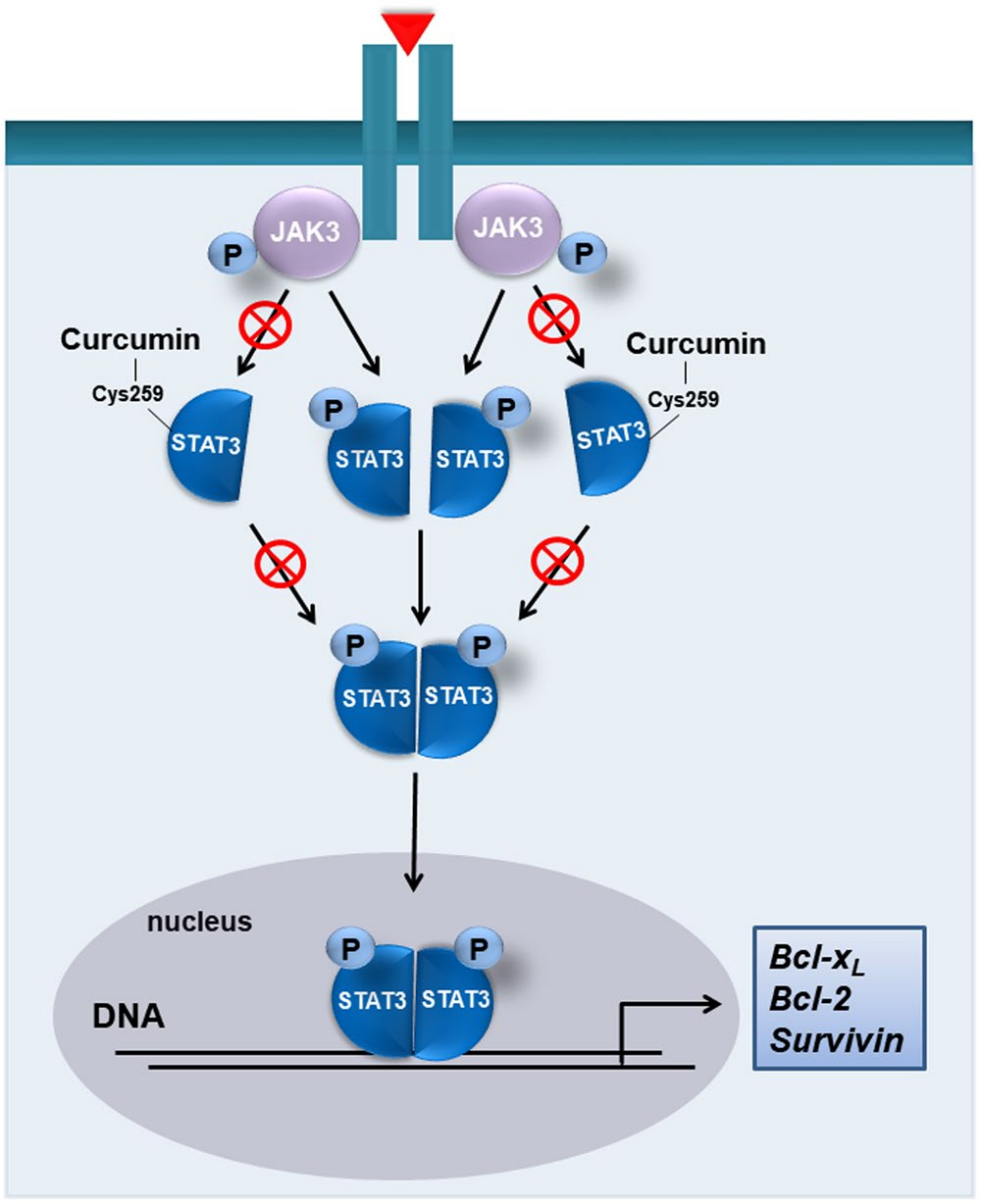
A proposed mechanism underlying the inactivation of STAT3 by curcumin. Covalent modification of STAT3 at its cysteine 259 residue by curcumin interrupts phosphorylation, dimerization and nuclear translocation of STAT3 and consequently induces apoptotic death in H-*Ras* MCF10A cells.

Many studies have focused on targeting the SH2 or DNA binding domain of STAT3 to disrupt STAT3:STAT3 or STAT3:DNA interaction^5,29,30^. Interestingly, one study suggests the possibility that curcumin can directly interact with the SH2 domain of STAT3, thereby disrupting STAT3 dimerization^4^. However, targeting the dimer interface of STAT3 is practically challenging due to the planarity of the large surface area^30^. Mass spectrometry analysis and *in silico* modeling data identified cysteine 251 and 259 present in the coiled-coil domain of STAT3 as the putative binding sites of curcumin. Although DNA binding and SH2 domains are generally targeted by STAT3 inhibitors, we could not find sites modified by curcumin other than cysteine 251 and 259. Similar to our findings, Stattic, a non-peptidic STAT3 inhibitor, was found to react with cysteine 251, 259, 367 and 426 of STAT3  which are not located in protein-protein or protein-DNA interfaces^31^. Thus, it is plausible that cysteine 251 and 259 residues to which curcumin predominantly binds are allosteric modification sites. We then further confirmed the specificity of the curcumin binding. It was observed that Sepharose-conjugated curcumin bound to wild type and to a slightly lesser extent, cysteine 251-mutated STAT3, while cysteine 259-mutated STAT3 barely interacted with curcumin. Furthermore, curcumin-induced apoptosis in PC3 cells harboring cysteine 259-mutated STAT3 was abrogated compared with that in cells transfected with wild type or cysteine 251-mutated STAT3. These may suggest that cysteine 259 is more likely to be involved in curcumin-induced apoptosis than cysteine 251. Consistent with our findings, it was found that methyl-2-cyano-3,12-dioxooleana-1,9(11)-dien-28-oate (CDDO-Me) suppressed STAT3 dimerization by alkylating cysteine 259^32^. Therefore, cysteine 259 of STAT3 is considered essential in its function and a specific binding site of curcumin (Fig. 9).

Though cysteine 259 of STAT3 is found to be covalently modified by curcumin, this amino acid is known to be prone to oxidation. Thus, it has been reported that STAT3 forms a dimer via an interchain disulfide bond between cysteine 259 residues^19^, and this dimerization appeared to occur independently of tyrosine 705 phosphorylation. The functional significance of oxidation of cysteine 259 residue remains to be clarified.

At this moment, we cannot assure whether the inhibitory effects exerted by curcumin on STAT3 signaling are solely due to the direct interaction with this transcription factor. There are diverse effector molecules in STAT3-mediated cell signaling networks including kinases, tumor suppressors and phosphatases. They work coordinatedly or independently of one another. Therefore, curcumin may inhibit STAT3 overactivation through multiple mechanisms. It has been reported that curcumin can upregulate Protein Inhibitor of Apoptosis Signaling 3 (PIAS3) which is known as a STAT3 repressor^13^. However, given that cells with cysteine 259-mutated STAT3 did not exhibit the direct interaction with curcumin as well as significant induction of apoptosis upon curcumin treatment, this specific interaction may be dominant in inhibiting STAT3 signaling. The effects of curcumin on expression/activation of PIAS3 and other potential STAT3-associated signaling pathways merit further investigation.

## Methods

### Cells and reagents

Normal human mammary epithelial (MCF10A) and H-*Ras* MCF10A cells were grown in DMEM/F-12 supplemented with 5% horse serum, 100 ng/ml cholera toxin, 20 ng/ml human epidermal growth factor, 10 μg/ml insulin, 0.5 μg/ml hydrocortisone, 2 mM L-glutamine and 100 units/ml penicillin/streptomycin. Human prostate cancer (PC-3) cells were grown in RPMI 1640 supplemented with 10% fetal bovine serum, and P-STAT3-luc stably expressing human cervical cancer (HeLa) cells were grown in Dulbecco’s modified Eagle’s medium (DMEM). Curcumin was purchased from LKT Laboratories, Inc. (St. Paul, MN, USA). Tetrahydrocurcumin was prepared by catalytic hydrogenation of curcumin. DTT and NAC were purchased from Sigma-Aldrich (St. Louis, MO, USA). Recombinant human IL-6 and OSM were purchased from R&D Systems, Inc. (Minneapolis, MN, USA). Primary antibodies for P-STAT3^Y705^, STAT3, HA-tag (anti-rabbit), Myc-tag (anti-mouse), and 10× lysis buffer were purchased from Cell Signaling Technology (Danvers, MA, USA). Primary antibodies against P-STAT3^Y705^, STAT3, phosphorylated extracellular signal-regulated protein kinase (P-ERK) and phosphorylated JAK3 (P-JAK3) were purchased from Santa Cruz Biotech (Santa Cruz, CA, USA). Primary antibody against STAT3 was purchased from BD Biosciences (San Hose, CA, USA). FITC-conjugated Annexin v. STAT3 recombinant protein was purchased from Active Motif (Carlsbad, CA, USA).

### RNA interference-mediated knockdown

STAT3 knockdown was achieved using STAT3 siRNA with the following target sequence: sence: 5′-CUAUCUAAGCCCUAGGUUUdTdT-3′; antisense: 5′-CCUAGGGCUUAGAUAGdTdT-3′.

### Immunoprecipitation and Western blotting

H-*Ras* MCF10A cells were treated with vehicle, curcumin or tetrahydrocurcumin for 12 h and digested with lysis buffer. Protein A/G agarose beads were washed with the same lysis buffer 5 times. The cell lysate of each group was immunoprecipitated with protein A/G agarose beads and STAT3 antibody (Santa Cruz Biotech) at 4 ${\rm{^\circ }}{\rm{C}}$ overnight using a rotator. PC-3 cells were transiently co-transfected with Myc-tagged STAT3 and HA-tagged STAT3 vectors. Cells were then treated with DMSO, curcumin or tetrahydrocurcumin for 12 h. Myc-tagged STAT3 was immunoprecipitated with A/G agarose beads and Myc-tag antibody and protein was detected by HA-tag antibody.

### Chromatin Immunoprecipitation (ChIP) assay

Chromatin prepared from H-*Ras* MCF10A cells was immunoprecipitated with anti-STAT3 antibody. The binding of STAT3 at promoter regions of Survivin, Bcl-2 or Bcl-xL was detected using primers with the following sequence. Survivin forward primer, 5′-CAGTGAGCTGAGATCATGCC-3′; reverse primer, 5′-TATTAGCCCTCCAGCCCCAC-3′. Bcl-2 forward primer, 5′-CTTCATTTATCCAGCAGCTT-3, reverse primer, 5′-GAGGGGACGATGAAGGAG-3′. Bcl-xL forward primer, 5′-CTGGGTTCCCTTTCCTTCCA-3′, reverse primer, 5′-TCCCAAGCAGCCTGAATCC-3′.

### Luciferase reporter gene assay

HeLa/P-STAT3-luc reporter cells were cultured (5 × 10^4^) in a 6 well plate and pretreated with curcumin for 12 h and then stimulated with 10 ng/ml oncostatin M for 6 h. The cells were lysed with a reporter lysis buffer (Promega, Madison, WI, USA), and the cell lysate (20 μl) was gently mixed with 100 μl luciferase assay reagent (Promega). The luciferase activity was measured using a luminometer (AutoLumat LB 953, EG&G Berthold, Bad widbad, Germany).

### Molecular modeling

AutoDock Vina program^33^ (The Scripps Research Institute, CA, USA) was used to make a docking model of curcumin binding to STAT3 protein. The structures of STAT3 are from Protein Data Bank (PDB ID: 3CWG and PDB ID: 1BG1). Curcumin coordinates were obtained from the GlycoBioChem PRODRG2 Server (http://davapc1.bioch.dundee.ac.uk/prodrg/)^34^. The grid maps for the docking model was set to have 74 × 126 × 126 points with 1.0 Å spacing to cover all surface of STAT3. Other parameters were set in a default value in AutoDock Vina program. Binding mode analysis of curcumin was carried out using a covalent docking module implemented in Maestro program v9.5 (Schrödinger LLC, NY). The STAT3 crystal structure (PDB code: 1BG1) was downloaded from PDB bank. STAT3 protein was prepared with neutralization and energy minimization using ProteinPrep Wizard. Curcumin was prepared with protonation at pH 7.4 and energy minimization using LigPrep module. In a covalent docking module, the reaction type of ligand was set to Micheal addition with cysteine 259 of STAT3 protein. Grid box was generated with residues within 5.0 Å of the cysteine 259. The 10 initial output of covalent binding pose was energetically minimized with residues within 3 Å of curcumin, and their binding energy was calculated by Prime MM-GBSA module. The covalent binding pose of curcumin was selected with low PrimeΔG_bind_ for binding mode analysis.

### Anchorage independent growth by soft agar assay

Underlayers consisting of DMEM/F-12 media containing 0.5% agar in 60 mm^2^ plates were prepared. H-*Ras* MCF10A cells (1 × 10^5^) were added to media containing 3.3% agarose and layered onto the previously prepared agar plates. DMSO, curcumin or tetrahydrocurcumin was given every 3 days. The colonies were allowed to grow for 3 weeks and then detected under a microscope.

### Generation of CNBr-activated-Sepharose-4B-conjugated curcumin and tetrahydrocurcumin

To generate CNBr-activated-Sepharose-4B-conjugated curcumin and tetrahydrocurcumin, Sepharose-4B beads (GE Healthcare, Piscataway, NJ, USA) were activated with 1 mM HCl. The activated beads were transferred into a 15 ml conical tube containing coupling buffer (0.1 M NaHCO_3_/0.5 M NaCl in 40% DMSO) and curcumin or tetrahydrocurcumin and rotated overnight at 4 °C. The beads were then washed with the same coupling buffer 5 times and again incubated with 0.1 M Tris.HCl overnight. After overnight incubation, beads were rinsed with a buffer containing 0.1 M acetate/0.5 M NaCl and a buffer containing 0.1 M Tris.HCl/0.5 M NaCl 3 times each and then with PBS.

### Cell-based pull-down assay

Curcumin-conjugated Sepharose-4B and tetrahydrocurcumin-conjugated Sepharose-4B beads were incubated with the lysates of H-*Ras* MCF10A cells overnight. The beads were then washed with a washing buffer (1 M Tris, 0.5 M EDTA, 1 M NaCl, 1 M DTT, 10% NP-40, 10 mg/ml BSA, 0.1 M PMSF, proteinase inhibitor cocktail) 5 times. The protein was detected with anti-STAT3 antibody (Cell Signaling Technology). PC-3 cells were transfected with GFP-tagged wild type STAT3, GFP-tagged C251A STAT3 or GFP-tagged C259 A STAT3 vector and lysed with a 1×lysis buffer. The cell lysates were precipitated with curcumin-conjugated Sepharose 4B beads. The protein was detected with anti-HA antibody.

### FACS analysis

Induction of apoptosis was measured by flow cytometry. H-*Ras* MCF10A cells were seeded in a 6 well plate and treated with curcumin or tetrahydrocurcumin for 12 h. Cells were permeablilized with 0.1% Triton X-100 and stained with FITC-conjugated annexin v and PI. FITC positive cells were detected using a FACSCalibur flow cytometer (BD Biosciences).

### Mass spectrometry sample preparation

The recombinant STAT3 protein (Active Motif) was treated with curcumin, and the solution mixture was added to the urea buffer (1 M urea, 50 mM Tris-HCl, pH 7.8). The sequencing grade modified trypsin (250 ng/μl) was then added to the above mixture for the digestion of protein followed by incubation at 37 °C for 8 h. The resulting tryptic peptides were desalted using Pierce® C-18 spin columns (Pierce Biotechnology, IL, USA). Thereafter, they were dried using Speed-Vac and then reconstituted in 200 μl of mobile phase for LC-MS analysis.

### LC-MS/MS analysis and database search

The HPLC analysis was performed on Easy-nLC 1000 system (Thermo Fisher Scientific Inc., Germany) equipped with a trap column (C18, 75 μm $\times$ 2 cm, 5 μm, Thermo Fisher Scientific Inc.) for cleanup followed by an EASY-Spray column (C18, 75 μm $\times$ 50 cm, 2 μm, Thermo Scientific Inc.). The separation of peptides was achieved by using the mobile phase comprising of 0.1% formic acid in water (Solvent A) and 0.1% formic acid in acetonitrile (Solvent B) in a gradient elution mode with a total run time of 90 min. The optimized gradient elution program was set as follows: (T_min_/% solution of B): _0_/5, _60_/50, _61_/80, _70_/80, _72_/1, _90_/1. The flow rate of the mobile phase was increased gradually throughout the gradient run (0–60 min: 200 nl/min; 61–71 min: 300 nl/min and 72–90 min: 350 nl/min). The column temperature was maintained at 60 °C and the injection volume was 10 μl. For LC-MS analysis, Easy-nLC 1000 system (Thermo Fisher Scientific Inc.) was coupled to Q-Exactive mass spectrometer (Thermo Fisher Scientific Inc.) equipped with an EASY-Spary^TM^ source. The data acquisition was under the control of Xcalibur software. The typical operating source conditions for MS scan in positive mode were optimized as follows: spray voltage, 2.1 KV; capillary temperature, 320 °C; and nitrogen was used as damping gas. All the spectra were recorded under identical experimental conditions, and are average of 20–25 scans. The scan range was set from m/z 300–1800. For Higher energy Collision Dissociation (HCD) experiments, keeping MS1 static, the precursor ion of interest was selected using the orbitrap analyzer and the product ions were analyzed. The normalized collision energies used were 30 NCE. Resolution of precursor ions was set at 70,000 and was set at 17,500 for HCD experiments. The top 8 precursor ions in the MS scan were selected by orbitrap analyzer for subsequent MS/MS analysis. The raw data were analyzed using Thermo Proteome Discoverer 1.3 (Thermo Fisher Scientific Inc.) against STAT3 database (gi number; 47458820). The search parameters were optimized as follows: Precursor ion tolerance was set at 10 ppm, fragment ion tolerance was set at 0.8 Da, and two miss cleavage was allowed. Variable modification options were used for the modification of curcumin (+368.126 Da) and carbamidomethylation (+57.021) on cysteine and oxidation (+15.995 Da) on methionine.

### Statistical analysis

The data were presented as means ± SD. Difference between groups was analyzed by Student’s unpaired *t*-test. *P* values less than 0.05 were considered significant.

### Data availability

The data sets generated and/or analysed during this study are included in this publication and in its Supplementary Information file.

## Electronic supplementary material

Supplementary Information
