# Supplementary material for: Curcumin interacts directly with the Cysteine 259 residue of STAT3 and induces apoptosis in H-Ras transformed human mammary epithelial cells
Source: Sci Rep. 2018 Apr 23;8:6409. doi: 10.1038/s41598-018-23840-2 (PMC5913338; doi:10.1038/s41598-018-23840-2)
Supplement: Supplementary file 1 — Supplementary Information [file 41598_2018_23840_MOESM1_ESM.pdf]

- Supplementary Information -

Curcumin interacts directly with the Cysteine 259 residue of STAT3 and induces apoptosis in H-*Ras* transformed human mammary epithelial cells

[**Running title**: Curcumin directly binds and inactivates STAT3]

Young-Il Hahn[1], Su-Jung Kim[1], Bu-Young Choi[2], Kyung-Cho Cho[3], Raju Bandu[3], Kwang Pyo Kim[3], Do-Hee Kim[1], Wonki Kim[1], Joon Sung Park[1], Byung Woo Han[1], Jeewoo Lee[1], Hye-Kyung Na[4], Young-Nam Cha[5] and Young-Joon Surh[1]

[1]*Tumor Microenvironment Research Center and Research Institute of Pharmaceutical Science,, Seoul National University, Seoul 08826, South Korea.*

[2]*Department of Pharmaceutical Science and Engineering, School of Convergence Bioscience and Technology, Seowon University, Chungbuk 361-742, South Korea.*

[3]*Department of Applied Chemistry, Institute of Natural Science, Global Center for Pharmaceutical Ingredient Materials, Kyung Hee University, Yongin, 17104, South Korea*

[4]*Department of Food Science and Biotechnology, College of Knowedge-based Services Engineering, Sungshin Women's University, Seoul 02844, South Korea.*

[5]*Inha University, College of Medicine, Incheon, South Korea*

1 **MAQWNQLQQL DTRYLEQLHQ LYSDSFPMEL RQFLAPWIES QDWAYAASK**E SHATLVFHNL

61 LGEIDQQYSR **FLQESNVLYQ HNLRRIKQFL QSRYLEKPME IAR**IVARCLW EESR**LLQTAA**

121 **TAAQQGGQAN HPTAAVVTEK** **QQMLEQHLQD VRK**RVQDLEQ K**MKVVENLQD DFDFNYKTLK**

181 **SQGDMQDLNG NNQSVTRQKM QQLEQMLTAL DQMRRSIVSE LAGLLSAMEY VQKTLTDEEL**

C C

241 **ADWKR**R**QQIA CIGGPPNICL DRLENWITSL AESQLQTR**QQ IK**KLEELQQK** **VSYKGDPIVQ**

301 **HRPMLEER**IV ELFRNLMK**SA FVVER**QPCMP MHPDRPLVIK **TGVQFTTKVR** **LLVKFPELNY**

361 **QLK**IKVCIDK DSGDVAALRG SR**KFNILGTN TKVMNMEESN NGSLSAEFK**H LTLREQRCGN

421 GGRANCDASL IVTEELHLIT FETEVYHQGL KIDLETHSLP VVVISNICQM PNAWASILWY

481 NMLTNNPKNV NFFTKPPIGT WDQVAEVLSW QFSSTTK**RGL SIEQLTTLAE K**LLGPGVNYS

541 GCQITWAKFC KENMAGKGFS FWVWLDNIID LVKKYILALW NEGYIMGFIS KER**ERAILST**

601 **KPPGTFLLRF SESSKEGGVT FTWVEKDISG KTQIQSVEPY TK**QQLNNMSF AEIIMGYK**IM**

661 **DATNILVSPL VYLYPDIPKE EAFGK**YCRPE SQEHPEADPG SAAPYLKTKF ICVTPFIDAV

721 WK

Supplementary Fig. S1. Recombinant human STAT3 exposed to 25 µM of curcumin.

The amino acid sequence coverage obtained by LC-MS/MS is indicated in green colour.

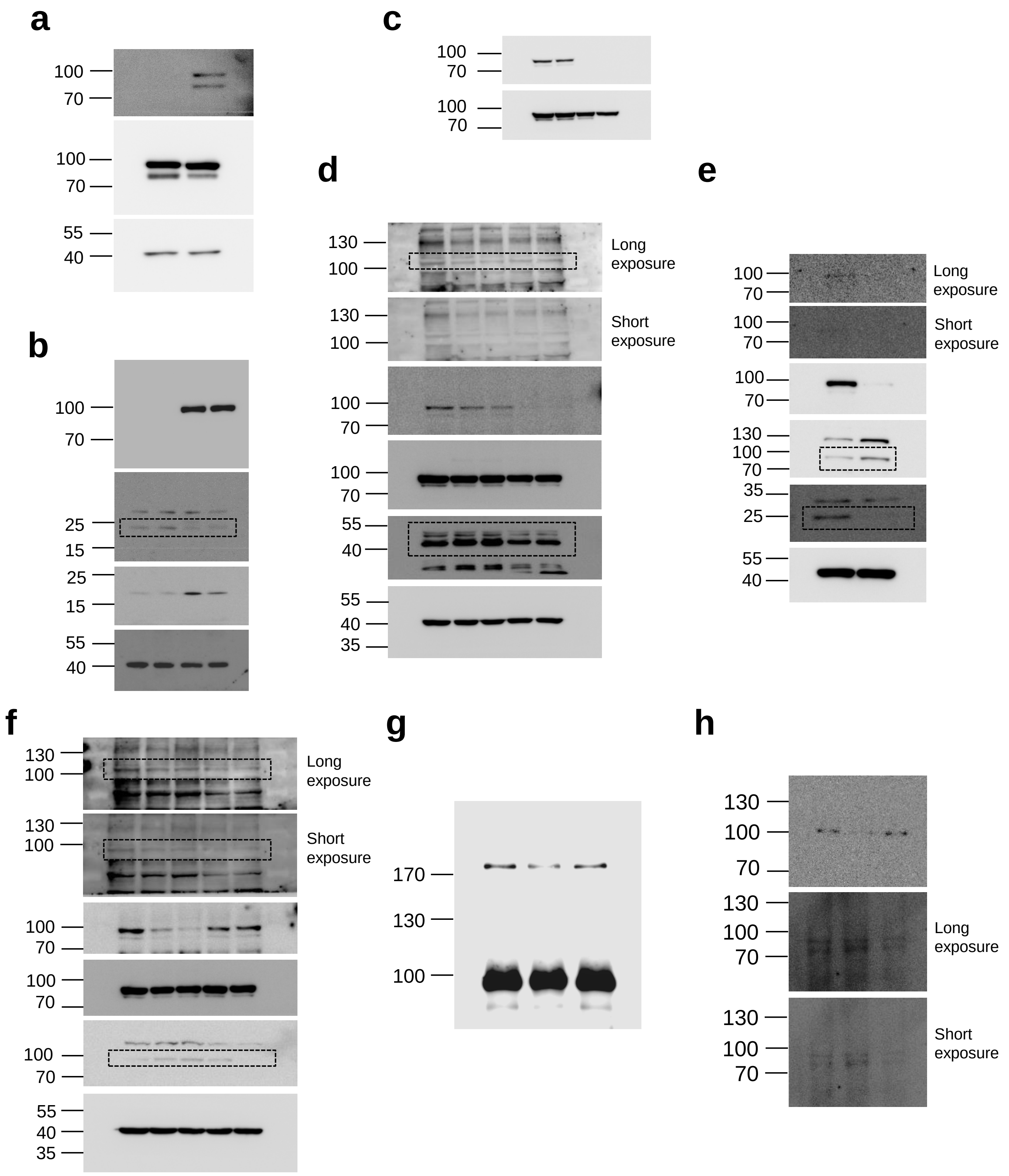

Supplementary Fig. S2. Full length blots corresponding to main figures. (a,b) Full blots corresponding to Fig. 1A. and 1D, respectively. (c,d,e) Full blots corresponding to Fig. 2A. 2C and 2D, respectively. (f) Full blot corresponding to Fig. 3B. (g,h) Full blot corresponding to Fig. 4A. and 4B, respectively. Indicated parts surrounded by a black line are shown in corresponding figures.

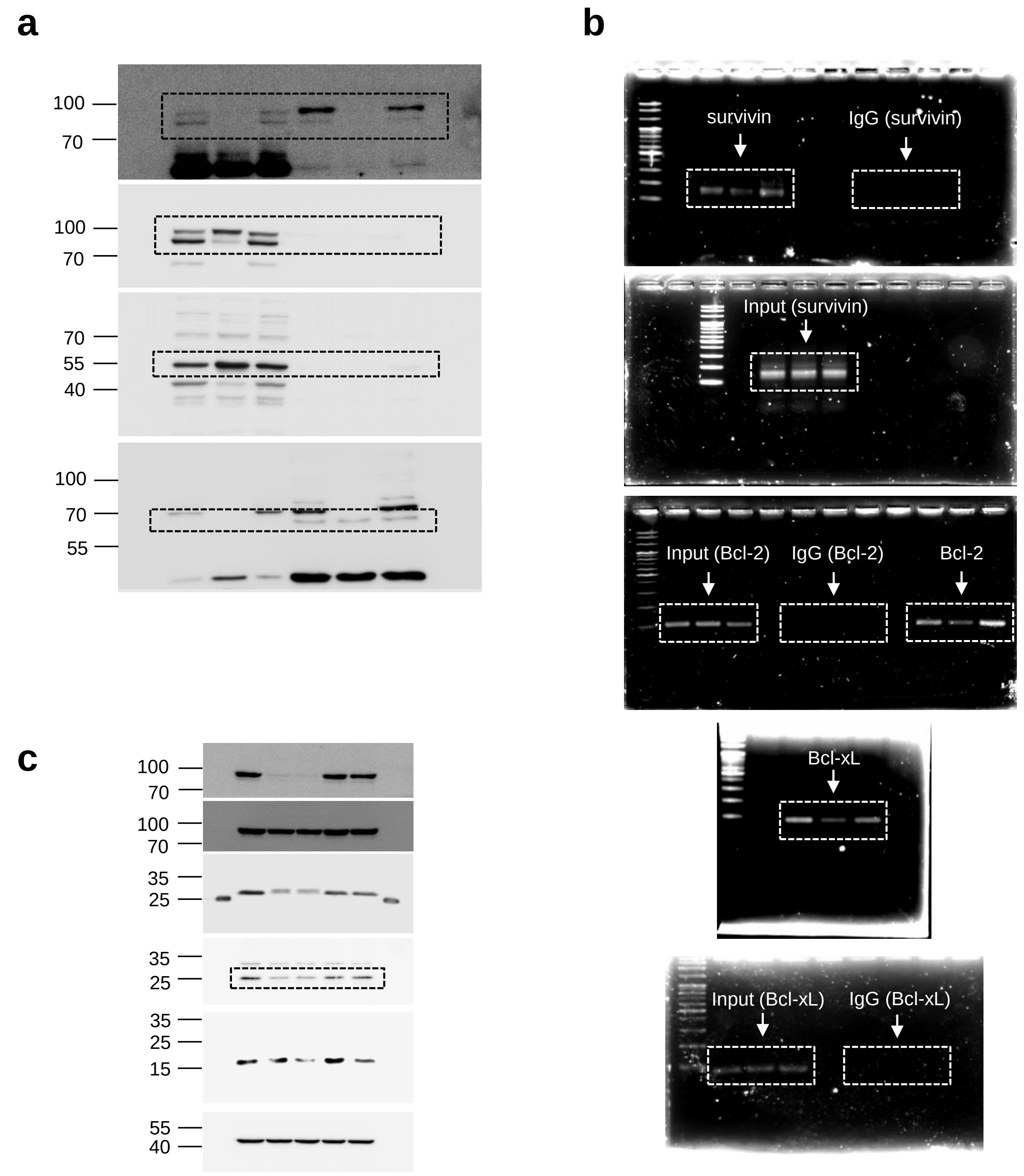

Supplementary Fig. S3. Full length blots corresponding to main figures. (a,b,c) Full blots corresponding to Fig. 5A. 5C and 5D, respectively. Indicated parts surrounded by a black line are shown in corresponding figures.

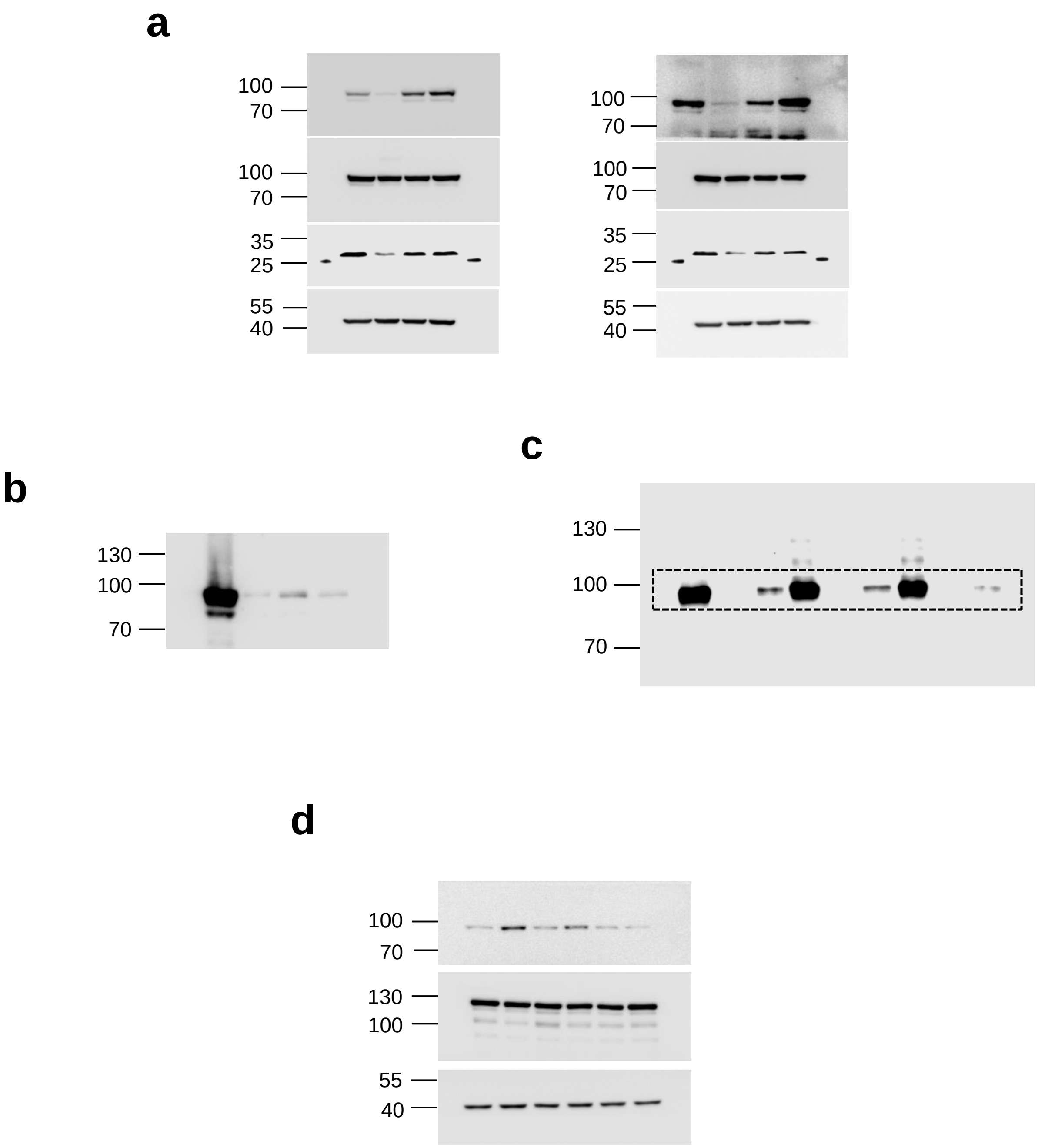

Supplementary Fig. S4. Full length blots corresponding to main figures. (a,b,c,d) Full blots corresponding to Fig. 6A. Fig. 8A, Fig. 8B and Fig. 8C, respectively. Indicated parts surrounded by a black line are shown in corresponding figures.
